# Refined Multi-Scale Mechanical Modeling of C/C-SiC Ceramic Matrix Composites

**DOI:** 10.3390/ma19010105

**Published:** 2025-12-28

**Authors:** Royi Padan, Chen Dahan-Sharhabani, Omri Regev, Rami Haj-Ali

**Affiliations:** School of Mechanical Engineering, Tel Aviv University, Tel Aviv-Yafo 6997801, Israel

**Keywords:** ceramic matrix composite (CMC), microstructure, micromechanics, representative volume element (RVE), PHFGMC, finite element analysis (FEA), multi-scale

## Abstract

This study introduces a refined multi-scale micromechanical framework for analyzing C/C-SiC ceramic matrix composites (CMCs) using a dedicated Parametric High-Fidelity Method of Cells (PHFGMCs). A three-level geometric model is constructed from scanning electron microscope (SEM) micrographs and computed tomography (CT) scans. Specialized dual micro-meso nested PHFGMCs are employed to accurately generate the effective properties and spatial distributions of local stress fields in the highly heterogeneous microstructure of an 8-harness C/C-SiC representative volume element (RVE). The proposed refined framework recognizes the different micro- and meso-scales, ranging from the carbon fiber and amorphous carbon matrix to intra-yarn segmentation and weave regions. All are nested in a complete 8-harness architecture. The refined PHFGMC analyses showed good agreement between predicted mechanical properties and experimental data for C/C-SiC. The model’s ability to resolve local spatial deformation in the complex microstructure of C/C-SiC CMCs is demonstrated. These findings highlight the need for a refined multi-scale analysis that captures microstructural complexity and constituent interactions influencing both macroscopic and local responses in C/C-SiC CMCs. The proposed PHFGMC-based framework provides a robust theoretical and computational foundation for the future integration of nonlinear and progressive damage models within C/C-SiC CMC material systems.

## 1. Introduction

Ceramic matrix composites (CMCs) play an important role in advanced aviation for maintaining structural stability above 1200 °C. Among CMCs, Liquid Silicon Infiltration (LSI) C/C–SiC stands out for its moderate production costs, making it ideal for single-use or short-exposure applications. Introduced by Krenkel and the German Aerospace Center (DLR) in the early 1990s, the LSI process yields a complex microstructure that confers relatively high damage tolerance [1,2]. The C/C-SiC’s final microstructure, which determines its final thermo-mechanical properties, can be tuned by selecting different fiber arrangements, varying the partial volume of phenolic resin, or altering the time-temperature profile during thermal processing [1,3]. Yet, controlling refined microstructure is advantageous only once the desirable microstructure can be defined. Hence, it is substantial to develop a refined approach to pre-simulate and evaluate the thermo-mechanical properties of a given microstructure. Developing a micromechanical-based numerical model is crucial for this purpose. Such effort is expected to contribute to a better understanding of material behavior and to reduce long-term, high-cost process development iterations for an enhanced C/C-SiC material.

The C/C-SiC is a multiphase material consisting of four constituent phases resulting from the multi-step LSI process. The first step is to manufacture a carbon fiber-reinforced plastic (CFRP) having a matrix with a high-carbon content. Next, the CFRP is pyrolyzed under an inert atmosphere (N2) at temperatures above 900 °C to convert the polymer matrix into amorphous carbon, resulting in a two-phase C/C material. As a result of that transformation, segmentation occurs within the carbon bundles (due to local shrinkage stresses exceeding the matrix’s tensile strength). The subsequent infiltration of molten silicon at about 1600 °C fills the segmented yarns’ capillaries and reacts with the carbon to generate the silicon-carbide matrix (evolving with residual silicon) [1,2,4]. Mechanical testing of the C/C-SiC CMC showed that it can be treated as a homogeneous material showing orthotropic mechanical properties [5,6]. However, these tests require a long and expensive effort. Alternatively, considering the phases’ properties and geometry, a micromechanical approach can be used.

Several analytical micro-mechanical models exist for the mechanical response of CMC [7,8,9,10]. However, these analytical methods describe a two-phase unidirectional fiber-matrix structure that may suit SiC/SiC, C/SiC, and oxide CMC. Still, they do not aim to describe the complicated structure of the C/C-SiC. Indeed, numerical method-based studies have been reported for the C/C-SiC properties. These studies use a repeated unit cell or representative volume element (RVE) to represent the material structure and apply micro-mechanical numerical methods to derive the C/C-SiC mechanical response [11,12,13,14]. The method of cells (MOCs) [15] is one of the first discretization methods for the micro-mechanics of composite materials. MOC discretizes a two-phase composite material with four rectangular subcells (three for the matrix and one for the fiber) while enforcing averaged periodicity conditions. The generalized method of cells (GMCs) extends MOCs to allow an unlimited number of subcells and phases. Hence, GMCs can be used for multiphase material, and as such, it has already been used to describe the mechanical behavior of some CMC materials [16,17]. The High Fidelity Method of Cells (HFGMCs) applies a second-order displacement formulation to capture coupling between tensile and shear stresses [18]. Pineda et al. [14] studied the difference between GMCs and HFGMCs in predicting C/C-SiC elastic properties and showed that the difference can be found mainly in the shear moduli. However, these methods are limited to rectangular subcell representations that must be parallel to the RVE coordinate system. This requirement makes it difficult to depict microstructures with complex geometries, as in the current case.

Finite element analysis (FEA) can capture complex geometries due to its isoparametric geometric mapping [19]. Over the last two decades, as computational capabilities have advanced, FEA methods have increasingly been adopted in micromechanical modeling. C/C-SiC FEA-based micromechanical models can be found in [11,12,13,20,21]. Another numeric method that addresses complex geometries is the Parametric High-Fidelity Generalized Method of Cells (PHFGMCs) [22,23,24]. This numerical method combines the numerically efficient HFGMC formulation with the ability to capture complex geometries using trilinear mapping. Hence, this method may be preferable for the current micromechanical computation task for C/C-SiC. While the HFGMC formulation can handle only an array of hexagonal subcells aligned with the RVE’s coordinate system [22], the parametric HFGMC uses a uniform parent domain to allow a more suitable subcell division [23]. Thus, it saves significant computational effort to describe curved-based geometries, as widely seen in continuous fiber-based composites.

Hence, the challenge of refined representation of C/C-SiC incorporates two main challenges. The first challenge is integrating all the phases’ properties along with the yarn’s segmented intra-layer microstructure into a homogenized material representation, while the other is following the yarns to reveal the 8HS-based C/C-SiC ply properties. Some early works described the yarns of the C/C-SiC as a group of carbon fibers arranged in a square-packed rectangular pattern embedded in the free-carbon matrix, modeling the SiC matrix as a thin layer wrapping the rectangular cross-section box from the outside [12,13,24]. Pineda et al. [14] showed the significance of applying more phases (free Si and voids) to the basic fiber-matrix description. These studies emphasized the importance of modeling all phases (in parallel or hierarchical models) to capture the combined effects of all encountered phases. These studies agreed well with the calculated homogenized properties and the referenced material’s elastic properties. Yet, they did not address the internal strain and stress field calculation, as needed for micro-mechanical-based damage evaluation prediction. Such meso-scale models have provided valuable insights into the design of carbon–epoxy composites [25] and oxide CMCs [26]; hence, this approach is also expected to contribute to the C/C-SiC case.

Generating a meso-(ply) 8-harness satin (8HS) geometric model is the second significant challenge for developing a comprehensive micro-mechanical model. Efforts to model an RVE of woven composites began decades ago [27,28] and have steadily progressed in capturing the detailed geometry and spatial paths of yarns within the weave. While these studies encompass various material systems such as glass-epoxy, carbon-epoxy, and oxide ceramic matrix composites (CMCs), they share a common feature: the yarns are encased in a surrounding matrix [26,29,30,31,32,33]. Thus, these studies define the yarn’s cross-sectional shape and path while assigning the remaining RVE volume to the matrix phase. Reconstructing the meso-scale geometry by extracting yarn shape and path while assuming the remaining volume is devoted to the matrix phase has been used in micro-CT-based studies [34,35]. Yet, this methodology does not apply to C/C-SiC composites. Unlike carbon-epoxy systems (and certain CMCs, such as C/SiC and oxide CMCs), the SiC matrix in C/C-SiC does not encapsulate the yarns but instead infiltrates them [3]. In other words, the C/C-SiC lacks the matrix’s resin pockets observed in other composite systems, which preclude an adequate representation of the C/C-SiC 8HS meso-structure. Few studies address this distinctive feature of C/C-SiC by incorporating it into their RVE models [21,24]. However, they introduced only a partial unit cell. Hence, there is a need to develop a refined approach capable of resolving micro- and meso-scale local displacement fields for the C/C-SiC material.

This study presents a multi-scale refinement framework for modeling the C/C-SiC CMC. This approach facilitates the evaluation of homogenized thermo-mechanical properties while resolving the local stress distributions within the RVE. Section 2 describes the investigated material and the methods to build corresponding RVEs. Section 3 briefly describes the PHFGMCs, the chosen numerical method for evaluating mechanical response. The resulting models, along with the evaluated mechanical response, are reported in Section 4. The manuscript ends with the conclusions in Section 5.

## 2. Material and Characterization

### 2.1. Material System

The C/C-SiC sample plate used in this study was manufactured by the LSI process [1], which involved three primary steps: green body warm pressing, pyrolysis, and siliconization. The plate’s layup comprised twenty 8HS fabric plies (polyacrylonitrile-based carbon fibers) arranged in a sequential 0°/90° symmetric and balanced configuration, where the 0° direction corresponds to the fabric’s longitudinal direction. The sample exhibited a net fiber volume fraction (VF) of approximately 59%, a density of 1.9 g/cm^3^, and an open porosity of 3.0%. The fiber VF of the C/C-SiC plate was calculated based on the initial weight of the carbon fibers and the total volume of the finished plate. Density and open porosity measurements were performed on a machined specimen in accordance with ASTM C373-14 [36].

Three specimen types were machined from the plate for C/C-SiC microstructural characterization. Specimen dimensions were selected to satisfy the geometric and resolution constraints of each characterization technique while ensuring representative capture of the relevant microstructural length scales. For micro-scale characterization, two cubes with a 4 mm edge length and two rods with a 3 mm diameter and a 6 mm length were investigated. For 8HS meso-structure characterization, a rectangular bar with a cross-section of 6.50 mm × 6.33 mm was machined.

### 2.2. CMC’s Microstructures Characterization

Defining the RVE of a composite material is a critical factor in micromechanical modeling. For C/C-SiC composites, this task is particularly challenging due to the three orders of magnitude difference in scale between the 8HS weave pattern (>8 mm) and the diameter of a single fiber (~7 μm). Between these extremes lies the material’s intra-layer microstructure. This scale disparity is also reflected in the tools used for microstructural characterization. Scanning electron microscopy (SEM), with its sub-micron resolution, is ideal for capturing the doubly periodic microstructure of the C/C phase. Nano-CT imaging is highly effective for depicting and quantifying the ~300 μm intra-yarn segmentation pattern, which consists of the SiC matrix and free Si structures encapsulating segments of the C/C phase. Lastly, micro-CT imaging excels in capturing the 3D 8-harness weave pattern (~10 mm) and the yarn path along the weave (~2 mm), making it the preferred method for evaluating the meso-scale inter-yarn RVE. The following paragraphs elaborate on the technical information describing the specimens’ characterization.

To characterize the 8HS structure, a micro-CT scan with a spatial resolution corresponding to a voxel size of approximately 10 microns was performed. The bar specimen was scanned using a stack protocol with six turns and 1184 projections per turn, operated at an accelerating voltage of 145 kV. The specimen was positioned so that its longitudinal axis aligned with the machine’s ‘z’ direction. This orientation resulted in a diagonal cross-section length of 9 mm, enabling the desired spatial resolution. Subsequently, a nano-CT scan with spatial resolution corresponding to a voxel size of approximately 1 micron was conducted to study the intra-yarn microstructure. Given the 3 mm size limitation of the nano-CT source apparatus, designated cylindrical specimens with a diameter of 3 mm were used. Moreover, using a specimen with a height of 3 mm allows avoiding the stacking protocol. To maximize the CT resolution, an accelerating voltage of 85 kV was employed, yielding a 400 nm focal spot size. Finally, for the C/C phase microstructure characterization, a Quanta 200FEG ESEM (Thermo Fisher Scientific, Waltham, MA, USA) was used. Two small cubes with a 4 mm × 4 mm cross-section were mounted in a Bakelite holder, then ground and polished using 1-micron monocrystalline diamond abrasives and a lubricant. These specimens were then scanned using a solid-state detector. Magnifications ranging from ×200 to ×8000 were utilized to capture the representative microstructure of the carbon fibers and the amorphous carbon matrix.

### 2.3. Methodology

**Figure 1:**
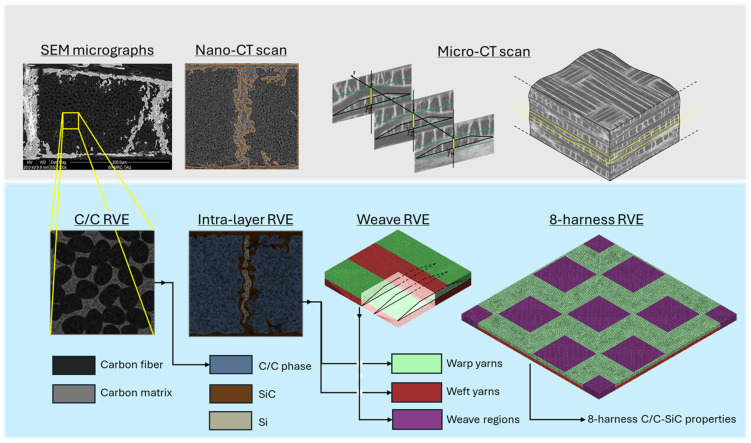
Microstructural multi-scale characterization for the specialized PHFGMC–CMC framework: microstructural characterizations (top row) and assembled RVE models (bottom row). Representative length scales range from the fiber diameter (~7 μm) through intra-yarn segmentation (~300 μm) to the 8HS weave pattern (~10 mm).

Accounting for the C/C-SiC differences in scale across micro- and meso-structures, a single microstructural scheme (or RVE) cannot adequately capture all the details while delivering effective, accurate solutions. Hence, a multi-level hierarchical approach was addressed (Figure 1). The following sections describe the method used to determine the RVE microstructure and its corresponding model at each level of this multi-scale structure. For clarity, this framework is described top-down for the RVEs construction method and bottom-up in the results section. For the phase properties, the current study adopted linear-isotropic and transversely isotropic formulations (depending on the specific phase). Thus, the suggested model does not consider features that affect damage and fracture, such as interfacial properties or woven-fabric imperfections. Nevertheless, the proposed framework is capable (for ongoing research) of introducing damage- and fracture-related properties, including interphase, interlaminar, and nonlinear properties, for each phase.

#### 2.3.1. 8HS Inter-Layer Meso-Scale RVE

**Figure 2:**
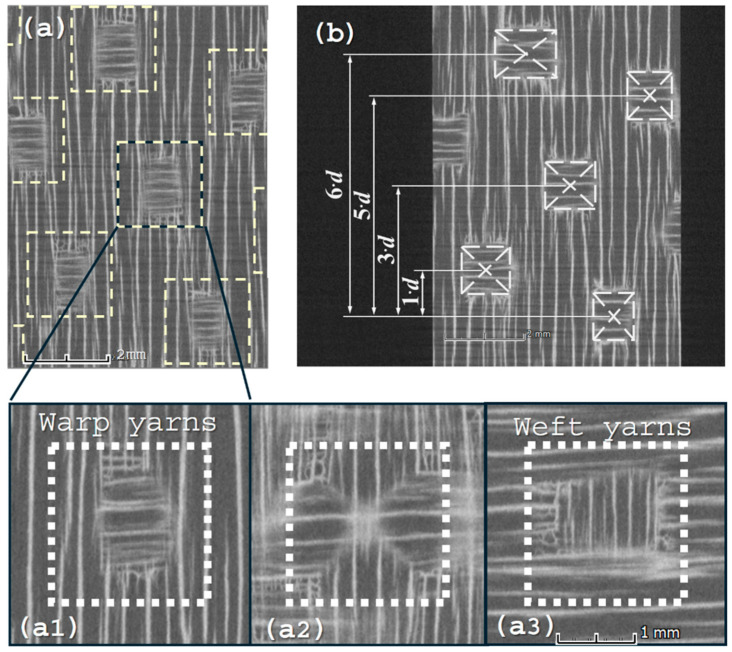
CMC with 8-harness ply CT images: (**a**) An overall scanned image; (**a1**) warp yarns layer cross-section; (**a2**) mid-ply cross-section revealing the maximum weave area; (**a3**) weft yarns layer cross-section; and (**b**) illustrating the measuring technique for distances between weaves.

**Figure 3:**
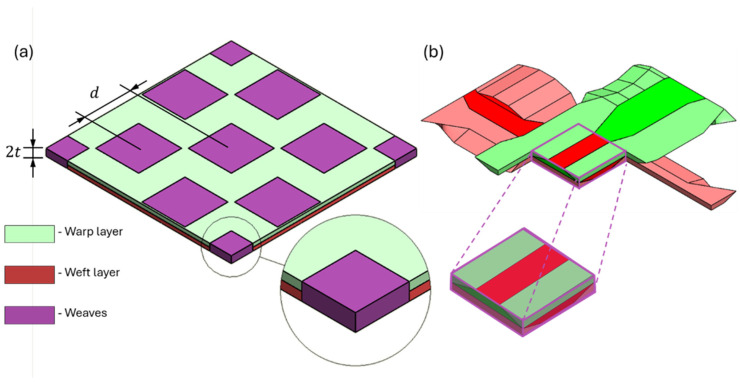
An idealized depiction of the 8-harness RVE nested model: (**a**) three-region decomposition, (**b**) weave region model.

The CT cross-section (taken from the bar micro-CT scan), shown in Figure 2, illustrates the 8HS satin structure as comprising two layers of perpendicular unidirectional fibers interspersed with ‘islands’ of interlacing yarns from both layers. Hence, a nested model is considered for the 8HS inter-layer meso-scale RVE. This nested RVE can be decomposed into a three-region structure: (i) warp yarns, (ii) weft yarns, and (iii) the weave region where the weft and warp yarns interlace (as seen in Figure 2a1, a2, and a3, respectively). Two parameters need to be determined for the RVE model (Figure 3): *d_avg_*—the averaged weave distance, and *t*—a single yarn layer thickness (where 2t corresponds to a single ply thickness). Both parameters are derived from the CT scan data. Calculating *t* is relatively straightforward; ten thickness measurements are taken across different cross-sections of the specimen to derive the average thickness. This value is then divided by the total number of plies and further halved to obtain the single-yarn-layer thickness. To calculate *d_avg_*, multiple cross-sectional images (one for each ply) are extracted from the CT scan and imported into CAD software (SolidWorks 2024). Using the CAD, the center point of each weave is determined based on its bounding rectangle (Figure 2b). Following that, the distances between different weaves are measured. Finally, *d_avg_* is calculated as a weighted average according to Equation (1), with the weighting designed to account for the number of weave distances in each measurement, thereby minimizing random errors.

(1) $$d_{avg}=\frac{\sum_{i=1}^{n}w_{i}\cdot d_{i}}{\sum_{i=1}^{n}w_{i}}$$

**Figure 4:**
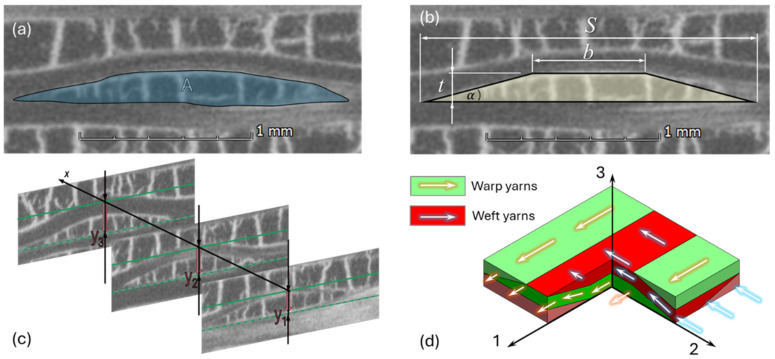
Weave region microstructural characterizations: (**a**) micro-CT scan of a typical cross-section of a woven yarn; (**b**) approximation of the yarn’s cross-section using a trapezoid shape along with main geometrical parameters; (**c**) defining the yarn path within multiple CT sections and associated measurements; (**d**) buildup of weave geometrical model to be nested in the 8HS PHFGMC-RVE.

The next step for the nested RVE model is defining the model representing the weave section. To that goal, the path and cross-section of the interlacing yarns need to be determined. A preliminary survey concluded that the hazel shape of the yarn’s cross-section should best approximate a trapezoid shape (Figure 4a and Figure 4b, respectively). This unique trapezoidal approximation allows the best fit of the yarn’s shape, but, more importantly, it prevents the formation of free volume between a yarn and the near-interlacing yarns. For carbon-epoxy RUCs, this volume (as would result from using any other convex shape) is considered to be filled with epoxy. However, such volume (often referred to as resin pockets) is not observed in C/C-SiC, where the ceramic matrix fills the segmentation within the yarn rather than lying between yarns.

Trapezoidal shapes are typically characterized by the height and lengths of the two bases. However, these values are not measured directly from the CT image in this study. In contrast, the height and base lengths are determined using more robust, less sensitive measurements to mitigate errors associated with contrast and resolution. First, the calculated value *t* is used for the trapezoid height. The trapezoid’s longer base dimension (*S*) is determined using the span of the perpendicular interlace yarn weave path. Taking advantage of the fact that the weaved-yarn section is coupled with the path of the perpendicular interlacing yarn. Hence, the yarn’s weave path is identified by recording its vertical coordinate at each CT section increment (Figure 4d), which corresponds to a 10 μm distance in the current scan. The points where the yarn path diverges from the neighboring yarns in the same layer are identified to determine the span (*S*). An average span value (*S_avg_*) is calculated using measurements from ten different yarn paths. The shorter base dimension (*b_avg_*) is calculated from the cross-sectional area (A) measured in CAD software. Measurements from 10 different images are averaged to compute bavg using Equation (2). Finally, the yarn’s inclination angle (α) is calculated using Equation (3). This angle ensures the correct orientation of the yarn’s orthotropic properties during PHFGMC calculations (Figure 4d) and facilitates qualitative comparisons of the stiffness between different woven satin configurations.

(2) $$b_{avg}=\frac{{2A}_{avg}}{t}-S_{avg}$$

(3) $$\alpha =\tan^{-1}\left(\frac{t}{S-s}\right)$$

#### 2.3.2. Intra-Layer Meso-Scale RVE

The intra-layer RVE represents the second level of microstructural modeling within the hierarchical framework. This level’s RVE is designed to capture the orthotropic properties of the yarn. To that goal, a single averaged intra-layer segment is used, following [13,14,20], rather than an entire yarn cross-section model. The high aspect ratio between the yarn’s length and the segment cross-section justifies the use of a 2D doubly periodic scheme for this intra-layer segment RVE. At this scale, the RVE is treated as a three-phase system, with the carbon/carbon (C/C) region modeled as a single homogenized phase. Thus, the current-level RVE representation includes the C/C, silicon carbide (SiC), and free silicon (Si) phases. CT image segmentation was performed using a geometry-driven, semi-manual CAD-based procedure. Automated grayscale thresholding was avoided because grayscale values do not uniquely correspond to individual phases in the multi-phase C/C–SiC system.

**Figure 5:**
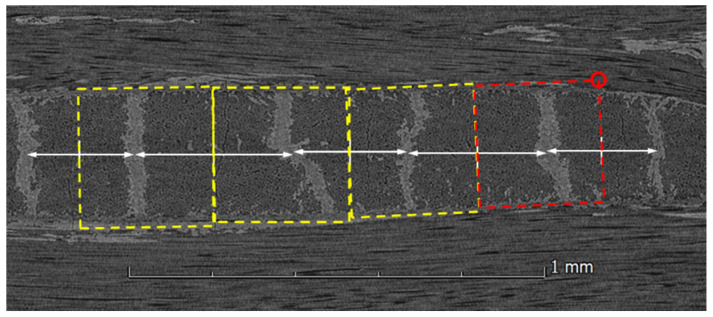
Construction process of an intra-layer RVE: candidates are illustrated in dashed rectangular borders. Three valid rectangles on the left and one invalid red rectangle on the right (arrows used to define the center point between segmentations; red circle indicates a vertex that falls out of SiC matrix, hence disqualifying the red rectangle).

The method to describe the rectangular RVE in this study diverges from the conventional approach, where the yarn’s RVE is typically modeled as a rectangular C/C region surrounded by a SiC matrix. The micro-CT scan survey reveals a significant distinction between the inter-layer SiC phase and the perpendicular (“vertical”) segmentation regions. The survey shows that while the inter-layer SiC phase aligns roughly parallel to the layer, the “vertical” SiC regions exhibit nonlinear, sloping boundaries. Given that these characteristics may affect out-of-plane stiffness, the RVE in this study incorporates the actual oblique geometry of the SiC matrix and the surrounding free Si inclusions. Modeling the RVE with the SiC segmentation centrally positioned allows it to capture the RVE’s oblique shape while satisfying periodic boundary conditions (Figure 5).

Identifying the appropriate CT cross-section for the 2D doubly periodic rectangular RVE involves several systematic steps. First, a survey is conducted across the scan’s cross-sections to identify potential rectangular segments. The procedure for defining rectangular borders involves several steps. First, straight lines are drawn that cross at the mid-height of each segmented area (Figure 5). Then, a rectangle is constructed with two edges passing through the midpoints of these lines. Finally, the rectangle orientation and edge length are determined by ensuring all four corners remain within the SiC matrix. Cross-sections meeting these criteria are deemed eligible candidates to serve as this level’s RVE. Each candidate is characterized by two key parameters: (i) a material property, specifically the constituents VF, and (ii) a geometrical property, defined as the width-to-height aspect ratio. Average values for these parameters are computed, and the candidate cross-section with properties closest to the averages is selected as the basis for the intra-layer RVE. This cross-section was then imported into CAD software, where C^2^ splines are utilized to precisely define the internal boundaries between the different phases, resulting in the final three-phase (segment-based) intra-layer RVE.

#### 2.3.3. C/C Micro-Scale RVE

The micro-scale C/C part of the RVE represents the lowest level in the hierarchy of the C/C-SiC multi-scale model. This RVE should characterize the distribution of carbon fibers within the surrounding amorphous carbon matrix. Square areas containing approximately ten fibers were selected as RVE candidates to identify the region that best describes the C/C microstructure. This fiber count balances the need to capture diversity in fiber shapes and arrangements while maintaining computational efficiency in the resulting RVE. The primary criterion for determining the representativeness of an RVE candidate is the apparent VF within its square boundaries, ensuring that the selected RVE fully represents the overall C/C phase for the higher-level modeling. Multiple square candidate regions are extracted from the SEM micrographs and imported into the CAD software. The fiber boundaries within each candidate region are manually traced using C^2^ splines, and the fibers’ volume fraction (VF) is calculated. The candidate having the carbon fiber’s VF closest to the average across all evaluated regions is selected to serve as the 2D doubly periodic RVE for the C/C microstructure.

#### 2.3.4. Simplified RVEs

Simplified RVEs have been shown to accurately predict effective properties [11,12,14]. Consequently, they are used as validation benchmarks for the proposed refined models. Additionally, they serve as a reference for assessing the proposed framework’s contributions to the prediction of local deformation fields. For the C/C micro-model, two simplified RVEs are analyzed: a square-packed RVE and a hexagonal-packed RVE. Both configurations are designed to match the refined RVE’s constituent volume fraction (VF). The intra-layer simplified RVE represents a symmetrical structure comprising two rectangular C/C phases separated by a SiC region that encapsulates free silicon (Si). For this level of RVE, both parameters used to pick the refined RVE (VF and the aspect ratio) are considered. Given that the high-level RVE representing the 8HS pattern is already idealized, no extra simplification is used for this level’s evaluation.

## 3. Dedicated CMC Extension of the Nested PHFGMC

**Figure 6:**
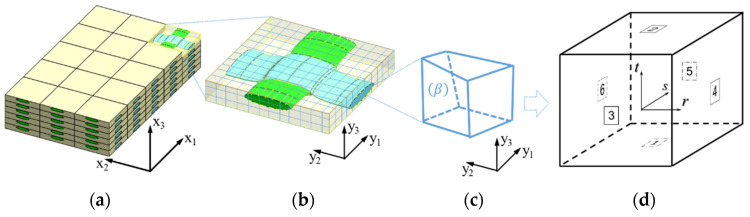
Typical meso-scale PHFGMC refined mesh: (**a**) an idealized composite material with triply periodicity described in a global coordinate system; (**b**) the 3D RVE represented in a local coordinate system; (**c**) an arbitrary quadrilateral subcell (β) represented in the PHFGMC element with its local coordinate system; (**d**) the uniform subcell in the parent domain and linear sub-parametric (r, s, t) coordinates.

The CMC’s mechanical properties and local deformation fields are determined using a micromechanical approach based on the PHFGMC. A comprehensive description of the 2D and 3D linear and nonlinear formulations is available in [31]. Here, a brief overview of the 3D formulation is provided. The PHFGMC geometric framework involves three distinct coordinate systems (Figure 6). The remote strains are defined on the full-scale composite in a global coordinate system ($x_{1}$, $x_{2}$, $x_{3}$)—see Figure 6a. The identified RVE (Figure 6b); however, is described with respect to a local coordinate system ($y_{1}$, $y_{2}$, $y_{3}$). The multi-phase RVE geometry is discretized into numerous single-phase quadrilateral subcells (Figure 6c). Each subcell is then mapped using conventional trilinear mapping, facilitated by standard shape functions, to a uniform subcell in the parent domain (Figure 6d), defined in a standardized parametric coordinate system (r, s, t). This mapping enables subcell-level calculations within a unified framework, enabling detailed analysis of the material’s mechanical behavior. Consequently, the displacement of any point within a certain cubic uniform subcell (*β*) in the parent domain is expressed using a complete quadratic form defined using ten micro-variables (*W_ijk_*). Using quadratic displacement expansion (Equation (4)) enables coupling between normal and shear deformation modes within each subcell while maintaining computational efficiency compared to higher-order isoparametric formulations.

(4) $$\begin{array}{c} u^{\left(\beta \right)}=u^{0}+W_{000}^{\left(\beta \right)}+W_{100}^{\left(\beta \right)}r+W_{010}^{\left(\beta \right)}s+W_{001}^{\left(\beta \right)}t+W_{110}^{\left(\beta \right)}rs+W_{101}^{\left(\beta \right)}rt+W_{011}^{\left(\beta \right)}st\\ +\frac{1}{2}W_{200}^{\left(\beta \right)}(3r^{2}-1)+\frac{1}{2}W_{020}^{\left(\beta \right)}(3s^{2}-1)+\frac{1}{2}W_{002}^{\left(\beta \right)}(3t^{2}-1) \end{array}$$

Seven independent equations are formulated for each (cubic uniform) subcell based on three governing conditions: periodicity and subcell displacement continuity, traction continuity, and force equilibrium. Since the quantity of micro-variables exceeds the number of equations, the bi-linear micro-variables are assumed to exhibit averaged behavior with respect to the corresponding quadratic terms (Equation (5)). This assumption ensures a computationally efficient framework for resolving the micro-variables while maintaining the model’s accuracy. Finally, ‘physical meaning’ micro-variables, related to the averaged displacements of subcell faces, are introduced to replace the original micro-variables while preserving the same coordinate system (Equation (6)).

(5) $$W_{110}^{\left(\beta \right)}=\frac{1}{2}\left(W_{200}^{\left(\beta \right)}+W_{020}^{\left(\beta \right)}\right)\;;\;W_{101}^{\left(\beta \right)}=\frac{1}{2}\left(W_{200}^{\left(\beta \right)}+W_{002}^{\left(\beta \right)}\right)\;;\;W_{011}^{\left(\beta \right)}=\frac{1}{2}\left(W_{020}^{\left(\beta \right)}+W_{002}^{\left(\beta \right)}\right)$$

(6) $$\begin{array}{c} {W_{k}^{\left(\beta \right)}\equiv \bar{u}}^{\left(\beta_{k}\right)}-u_{0}=\frac{1}{A_{k}}\int_{A_{k}}u^{\left(\beta \right)}\left(y\right)dA_{k}=\frac{1}{4}\int_{-1}^{+1}\int_{-1}^{+1}u^{\left(\beta_{k}\right)}\left(\xi ,\eta \right)d\xi d\eta ;\;k=1,\ldots ,6\\ W_{7}^{\left(\beta \right)}\equiv W_{000}^{\left(\beta \right)} \end{array}$$

Applying Equations (5) and (6) to Equation (4) leads to the overall displacement expression:

(7) $$\begin{array}{l} u^{\left(\beta \right)}={\;\varepsilon }_{0}\cdot x+W_{7}^{\left(\beta \right)}+\frac{1}{2}\left(W_{4}^{\left(\beta \right)}-W_{6}^{\left(\beta \right)}\right)r+\frac{1}{2}\left(W_{5}^{\left(\beta \right)}-W_{3}^{\left(\beta \right)}\right)s+\frac{1}{2}\left(W_{2}^{\left(\beta \right)}-W_{1}^{\left(\beta \right)}\right)t\\ +\frac{1}{4}(W_{4}^{\left(\beta \right)}+W_{6}^{\left(\beta \right)}-2W_{7}^{\left(\beta \right)})(3r^{2}+rs+rt-1)\\ +\frac{1}{4}(W_{3}^{\left(\beta \right)}+W_{5}^{\left(\beta \right)}-2W_{7}^{\left(\beta \right)})(3s^{2}+rs+st-1)\\ +\frac{1}{4}(W_{1}^{\left(\beta \right)}+W_{2}^{\left(\beta \right)}-2W_{7}^{\left(\beta \right)})(3t^{2}+rt+st-1) \end{array}$$

This expression establishes the connection between a linear remote deformation gradient at the macro scale ($\varepsilon_{0}\cdot x$) and the local perturbation displacement field at the micro-scale, described by a quadratic expression involving the subcells’ micro-variables (${W_{1}^{\left(\beta \right)}-W}_{7}^{\left(\beta \right)}$).

Periodic boundary conditions are inherently enforced in the PHFGMC framework, with remote loading applied via prescribed remote strains. In the common micromechanical problems, the remote strains are known, and the local strains (and stresses) should be calculated. However, in a more general case, even the remote strains can be treated as subcell variables, resulting in 27 unknown variables: 21 micro-variables ($W_{k}^{\left(\beta \right)}$) and six global variables ${\varepsilon_{0}}_{k}^{\left(\beta \right)}$. Consequently, for the PHFGMC algebraic formulation, the following three vectors can be defined as follows (Equations (8)–(10)):

(8) $${\varepsilon_{0}^{\left(\beta \right)}}_{\left(6\times 1\right)}=\;{\left\{\varepsilon_{11}^{\left(\beta \right)}\;,\;\varepsilon_{22}^{\left(\beta \right)},\varepsilon_{33}^{\left(\beta \right)}\;,\varepsilon_{12}^{\left(\beta \right)}\;,\varepsilon_{13}^{\left(\beta \right)}\;,\varepsilon_{23}^{\left(\beta \right)}\right\}}^{T}$$

(9) $${W^{\left(\beta \right)}}_{\left(21\times 1\right)}={\left\{W_{1r}^{\left(\beta \right)}\;,W_{1s}^{\left(\beta \right)},W_{1t}^{\left(\beta \right)},W_{2r}^{\left(\beta \right)}\;,\ldots \;,W_{7t}^{\left(\beta \right)}\right\}}^{T}$$

(10) $${{\hat{u}}^{\left(\beta \right)}}_{\left(27\times 1\right)}=\left\{W^{\left(\beta \right)}\;,\;\varepsilon_{0}^{\left(\beta \right)}\;\right\}={\left\{\varepsilon_{11}^{\left(\beta \right)},\;\varepsilon_{22}^{\left(\beta \right)}\;,\;\ldots \;,\varepsilon_{12}^{\left(\beta \right)},\;W_{1r}^{\left(\beta \right)},W_{1s}^{\left(\beta \right)},\;\ldots \;,W_{7t}^{\left(\beta \right)}\right\}}^{T}$$

where $\varepsilon_{0}^{\left(\beta \right)}$ is the remote strain tensor according to Voigt notation, $W^{\left(\beta \right)}$ is the subcell’s micro-variables vector, and ${\hat{u}}^{\left(\beta \right)}$ is the unknown variable vector (to be solved).

The current study uses a two-layer (local-global) averaged virtual work principle of the PHFGMC, as finalized in [31]. The virtual work formulation of the PHFGMC yields a symmetric stiffness matrix, effectively overcoming the computational challenges associated with the non-symmetric system of equations in the conventional HFGMC method. Consequently, this formulation facilitates the use of Newton’s methods for incremental, nonlinear solutions, which are essential for future modeling of damage within the material framework [33].

The average virtual work is expressed at two levels: (i) at the local (subcell) level in terms of face-average (virtual) displacement micro-variables associated with the average face tractions, and (ii) at the remote global strains introduced via a virtual work formulation associated with the average stresses. The equilibrium between the internal and external average virtual work of a specific subcell (β) is expressed by Equation (8). This equation is suitable for small deformation scenarios, such as those expected in the C/C-SiC system.

(11) $$\int_{V^{\left(\beta \right)}}\delta \varepsilon^{(\beta ),T}\sigma^{\left(\beta \right)}dV=\delta \varepsilon_{0}^{(\beta ),T}f_{U}^{\left(\beta \right)}+\delta W^{(\beta ),T}f_{W}^{\left(\beta \right)}$$

where $\varepsilon^{\left(\beta \right)}$ represents the symmetric portion of the total deformation gradient,$\;\sigma^{\left(\beta \right)}$ denotes the Cauchy stress tensor, and $f_{U}^{\left(\beta \right)}$ and $f_{W}^{\left(\beta \right)}$ are the external force vectors (related to the remote strains and subcell’s micro-variables), respectively.

The following is an explicit expression of each of these terms. First, the local strain vector (according to Voight notation, similar to Equation (5)) summarizes the remote strain and the local perturbation:

(12) $$\varepsilon^{\left(\beta \right)}=\varepsilon_{0}+B_{W}^{\left(\beta \right)}W^{\left(\beta \right)}$$

where ${B_{W}^{\left(\beta \right)}}_{\left(6\times 21\right)}$ is a strain-displacement micro-variable matrix. It holds the relevant displacement gradient components (expressed with parametric coordinates) and incorporates the conjugate Jacobian components of the parametric mapping.

The stress vector is calculated according to generalized Hooke’s law for infinitesimal strains, based on the single-phase material’s stiffness matrix $C^{\left(\beta \right)}$ (constant within each subcell):

(13) $$\sigma^{\left(\beta \right)}=C^{\left(\beta \right)}\varepsilon^{\left(\beta \right)}=C^{\left(\beta \right)}\left(\varepsilon_{0}+B_{W}^{\left(\beta \right)}W^{\left(\beta \right)}\right)$$

The resisting force vectors hold the stress values contribution according to the following:

(14) $${f_{U}}^{\left(\beta \right)}=\int_{V^{\left(\beta \right)}}\sigma^{\left(\beta \right)}dV\;\;f_{W}^{\left(\beta \right)}=\int_{V^{\left(\beta \right)}}B_{W}^{(\beta ),T}\sigma^{\left(\beta \right)}dV$$

Substituting Equations (12)–(14) into Equation (11) leads to a straightforward algebraic formulation, holding a symmetric stiffness matrix:

(15) Ku^=∫VBWTCBWdV∫VBWTCdV∫VCBWdV∫VCdV(β)W ε0(β)=fU fW(β)=f

By solving each subcell’s micro-variables, one can calculate the material’s effective stiffness matrix by summing the partial contributions of each subcell:

(16) $$C^{*}=\frac{1}{V_{total}}\sum_{\beta =1}^{N_{sc}}V_{\beta }C^{\left(\beta \right)}G^{\left(\beta \right)}$$

where $C^{*}$ is the composite’s effective stiffness matrix, $V_{total}$ is the RVE’s total volume, $V_{\beta }$ is the volume of a single subcell, $C^{\left(\beta \right)}$ is the stiffness matrix of the subcell’s uni-material, and $G^{\left(\beta \right)}$ is its strain concentration tensor defined according to the following:

(17) $${\bar{\varepsilon }}^{\left(\beta \right)}\equiv G^{\left(\beta \right)}\cdot \varepsilon_{0}$$

It should be noted that this 3D formulation of the PHFGMC, as shown in Equations (8)–(12), is general, thus allowing a 3D micromechanical analysis of RVEs with both triply and doubly periodicity, thereby significantly reducing the number of degrees of freedom compared to conventional finite element–based RVEs. This unique ability to provide a full 3D strain and stress solution for a 2D geometric model (representing a doubly periodic structure) contributes to the PHFGMC’s high computational efficiency. This PHFGMC characteristic is leveraged to facilitate specialized C/C and intra-yarn micro-mechanical modeling, offering a promising approach to advancing this study area.

## 4. Results and Discussion

A bottom-up, step-by-step calculation is carried out to determine the effective properties of C/C-SiC. The results are presented in a hierarchical sequence, leading to the final effective properties. The following sections describe the resulting RVEs at each level of the micro-to-meso models and the evaluation of the in situ mechanical properties. For the PHFGMC formulation, subcell discretization influences the results of local stress fields; therefore, the subcell discretization was refined to ensure geometric fidelity of phase boundaries and was further refined until additional refinement no longer altered the predicted effective properties. All results reported herein correspond to the refined subcell discretization.

### 4.1. Micro-Scale RVE for the C/C Phase 

**Figure 7:**
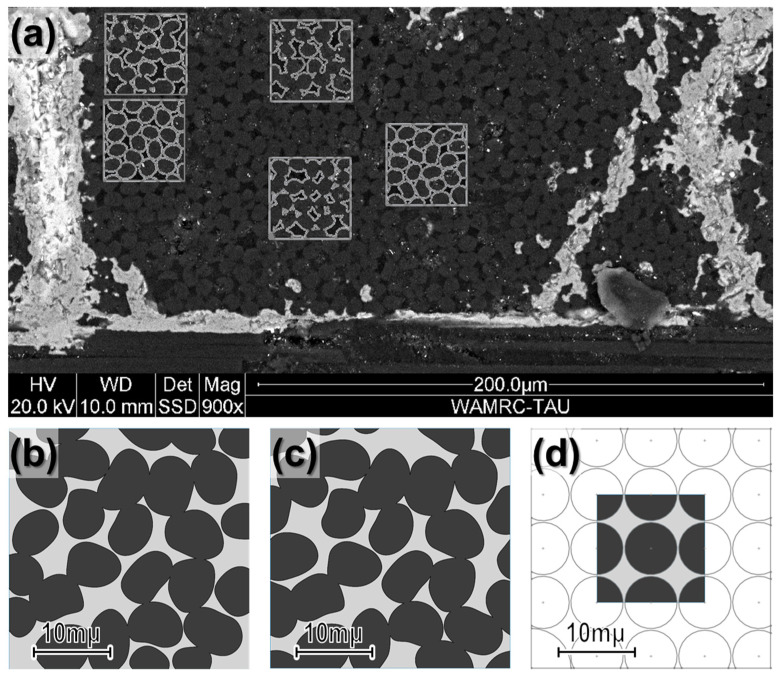
C/C SEM micrograph sample used to extract and define a refined C/C-RVE: (**a**) candidates for refined C/C-RVEs, (**b**) identified refined RVE, (**c**) idealized square-packed RVE, and (**d**) idealized hexagonal-packed RVE.

To calculate the average mechanical properties of the C/C, the RVE should contain at least ten fibers to capture the influence of the geometric arrangement (i.e., ten times larger than the common single fibers’ RUCs). Accordingly, a representative cross-sectional area measuring 32 µm by 32 µm was found to contain approximately ten complete fibers. Consequently, square models of this dimension were created to assess the VF of each phase (see Figure 7a). The analysis revealed a carbon fiber VF of nearly 74%. Therefore, the square model with the closest VF was selected for the refined model. As anticipated, the fibers within the RVE are not arranged in a defined pattern, and the resulting representative volume element (RVE) illustrated in Figure 7b is far from resembling the often-used simplified model. This RVE passed minor modifications to ensure geometric periodicity. The final RVE is displayed in Figure 7c. A simplified square RVE (Figure 7d) was generated to compare the refined RVE with the typical RUC used in the literature [14], featuring fibers with a diameter of 7 µm and preserving the volume fraction consistent with the survey results.

The mechanical properties of the phases required for the PHFGMC calculations were assumed based on [12]. These properties of the carbon fiber were used as reported. Still, for the amorphous carbon matrix, the Young’s modulus of the isotropic material was reduced by 3% to incorporate the measured porosity. Hence, the properties used in this study are detailed in Table 1. The PHFGMC calculations for both C/C RVEs resulted in similar mechanical properties, as summarized in Table 2.

**Figure 8:**
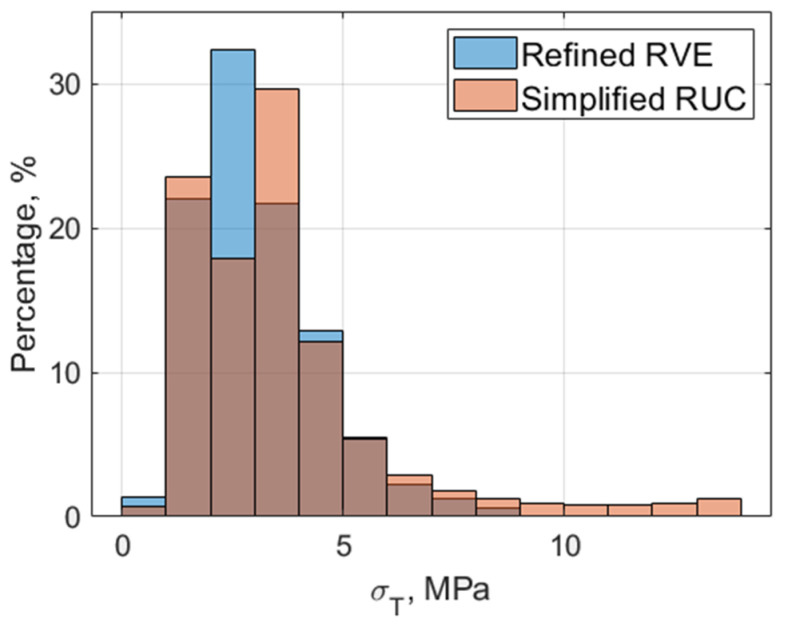
Carbon—matrix transverse stress distribution for the refined RVE vs. the simplified RUC (at a remote strain of $\varepsilon_{t}=0.001$).

Notably, there is good agreement in the elastic properties along the fiber direction (E_L_ and G_L_), with negligible differences across models. A larger difference is observed in the transverse direction, with a deviation of 8% for E_T_ and G_T_. The calculated stress fields disclose this difference. When subjected to remote longitudinal strain, the different models result in similar stress fields. For the transverse strain, the stress fields in the carbon matrix are influenced by microstructure diversity. Figure 8 shows that, for the simplified RUC case, the carbon matrix experiences higher strains (probably due to strain concentrations near the fiber and the close edge). These findings are consistent with previous reports on glass–epoxy composites [37] and carbon–epoxy composites [38,39,40]. While [39] attributes the difference to fibers’ longitudinal misalignment, refs. [38,40] focus on stress distribution in the matrix as the main cause of matrix failure. Since these two phenomena are not accounted for in the current doubly periodic model, this difference appears to be related to fiber shape and distribution. It is more likely that the lateral stiffness is sensitive to the lateral arrangement of the distinct phases across different RVEs.

These findings emphasize that when transverse properties are a concern, a simple geometric model is inadequate and may lead to over-exaggeration in the carbon matrix stress field. Underscores the necessity of employing a refined model to capture the actual impact of microstructural variations accurately.

### 4.2. Intra-Layer RVE

**Figure 9:**
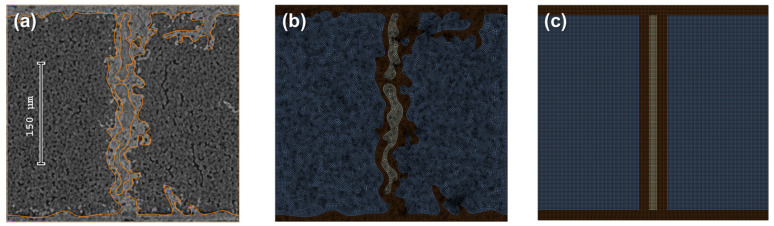
Intra-layer segment-RVEs. (**a**) CT image of a representative segmental section; (**b**) corresponding refined RVE; (**c**) simplified RVE.

CT data provides a 3D representation of the measured sample, allowing for the extraction of substantial information from a single scan. The nano-CT scans of the 3 mm rod specimens identified 15 eligible candidates from each specimen. These models were then analyzed to determine the segment’s aspect ratio (width-to-height) and the volume fraction of the three main phases considered at this scale of magnitude: C/C, SiC, and free Si. The current study did not account for cracks or pores within the C/C phase. Upon depicting the boundaries of the different phase constituents, special care was taken to determine whether specific inclusions within the segment model should be retained or excluded. An additional cross-section at a distance equal to the segment’s width was examined for verification. If the inclusion could not be identified in this adjacent cross-section, it was excluded from the segment model and the corresponding volume fraction calculations. This procedure demonstrates another advantage of creating a refined model based on CT scans. The survey results indicate an average phase VF of 80% C/C, 17% SiC, and 3% Si, along with an average width-to-height ratio of 1.07 (Table 3 details the average and range values for studied parameters). Accordingly, the cross-section closely matching these characteristics was selected as the current level’s RVE (Figure 9a,b). The simplified geometry RVE was generated to meet the refined RVE dimensions, with an identical aspect-ratio constituent VF characteristic (Figure 9c). Subsequently, four additional segments were analyzed to evaluate the sensitivity of the C/C-SiC effective properties to the observed tolerance range in the surveyed parameters. Two models with similar C/C VF (~82%) with low (1.0) and high (1.35) aspect ratios, and two models with similar aspect ratios (0.95) but different C/C VF (75% and 87%) were selected. In total, six models were analyzed: five refined models and one idealized RVE. For the intra-layer PHFGMC analysis, the resulting refined RVE properties (Table 2) were used for the C/C phase, while the SiC and Si properties were adopted from [14] (Table 4). These properties are adjusted values to those originally reported to ensure consistency with the as-fabricated C/C–SiC material system and with micromechanical modeling requirements. The primary sources underlying these properties include Technical Report AFML-TR-67-423 and Kelly (1973). The processed values reported by [14] are used here intentionally, as they provide a self-consistent property set appropriate for PHFGMC-based analyses.

The analysis revealed differences in the effective properties prediction for the different RVEs. The resulting properties for the refined RVE, simplified RVE, and RVEs with C/C minimum and maximum volume fractions are summarized in Table 5. For the simplified RVE, the main difference is observed in the Young’s modulus perpendicular to the layer (23.1 GPa vs. 20.8 GPa for the refined RVE). As for the C/C case, the excess stiffness in the transverse direction of the simplified RVE is also related to the lateral-ordered arrangement of the reinforcement phase, leading to an over-prediction of the elastic modulus in that direction. A strong dependence on the C/C phase VF was observed in the longitudinal properties, with E_L_ varying from 144.2 GPa for 73% C/C VF to 159.5 GPa for 84% C/C volume fraction. In contrast, the sensitivity to the RVE aspect ratio was very low; therefore, it is not presented.

**Figure 10:**
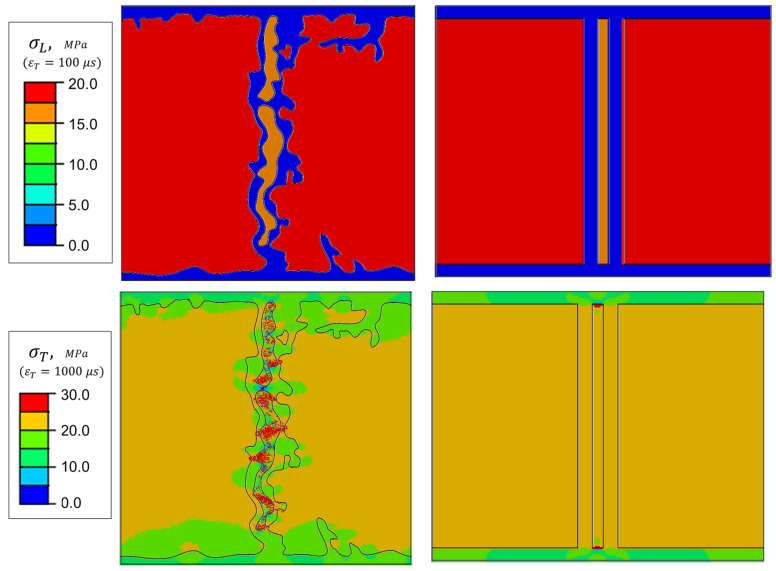

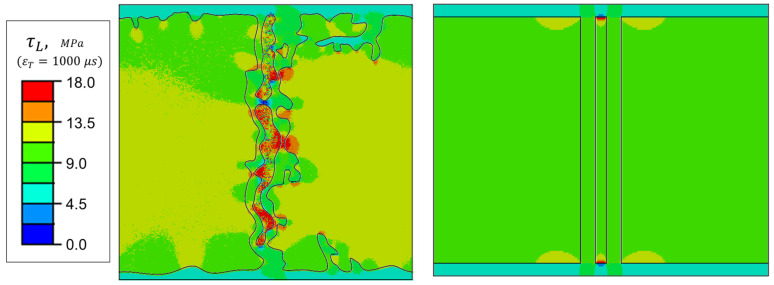
Stress-field contours: Simplified RVE vs. refined RVE for different applied remote strains. Longitudinal tensile strain (**top**), transverse strain (**middle**), and shear strain (**bottom**).

**Figure 11:**
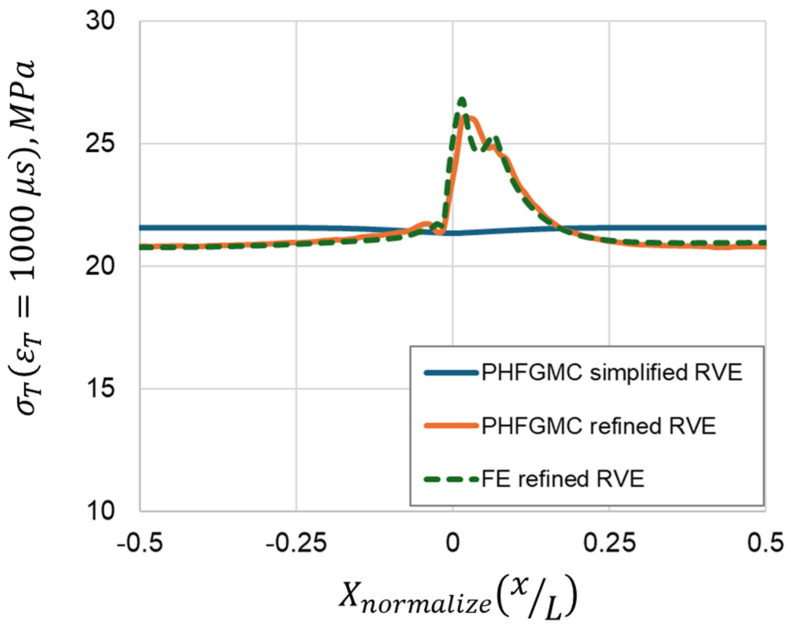
Edge-to-edge plot of the transverse normal stress under remote transverse strain for different RVEs and numerical models.

The PHFGMC analyses reveal the spatial distribution of stresses. Similarly to the sensitivity of the effective properties, it is evident that lateral stress fields are more sensitive to the actual microstructure. Figure 10 compares the transverse tensile stress (σ_T_) and τ_LT_ shear stress fields of the ideal RVE and the refined RVE under remote transverse train and shear strain, respectively. Significant differences in stress values and their spatial distribution are observed in both cases, particularly within the SiC region, which is of special interest given prior findings [41] indicating the role of SiC in overall toughness. Figure 11 presents the apparent transverse stress distribution in the midsection of both the intra-layer segment and the simplified RVE models. In addition, results from a finite element (FE) analysis of a 3D refined model are included to demonstrate the effectiveness of the specialized PHFGMC-CMC compared with a general-purpose displacement-based commercial code. The plot shows that differences in model phase boundaries lead to deviations in stress along the normalized x-axis. Notably, the idealized model predicts lower transverse tensile stress near the SiC boundaries and within the SiC and Si phases. Hence, the critical importance of employing a refined model is demonstrated.

### 4.3. Meso-Scale RVE of 8-HS Inter-Layer

For the 8-HS meso-scale, an idealized, relatively simple, and less refined model was generated due to the inherent complexity of the 8-HS weave. However, the current PHFGMC model accurately captures the weave architecture and geometry by incorporating detailed measurements from micro-CT scans, ensuring a faithful representation of the complex 8HS structure. The CT scan for the 8HS bar confirmed that, unlike carbon-epoxy structures, individual yarns in the C/C-SiC system could only be traced within the weave regions. The parallel yarns’ sections (following the pyrolysis and LSI process) appear as a single unit outside these regions. Thus confirming the approach of decomposition of the 8HS model into three distinct regions. All regions were modeled based on micro-CT scan measurements, resulting in an RUC with dimensions of approximately 8.6 mm × 8.6 mm × 0.16 mm. The weave-nested RUC, integrated into the ply model, represents the interlacing of the warp and weft yarns with an inclination angle of almost 15 degrees.

**Figure 12:**
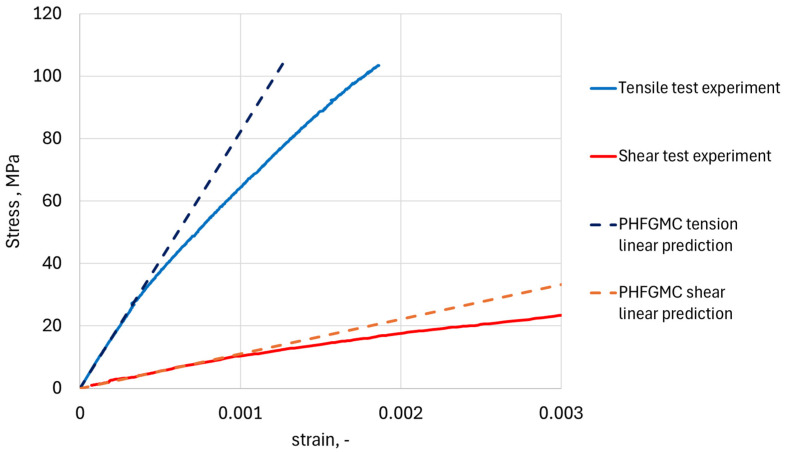
C/C-SiC experimental results along with the PHFGMC prediction of the linear, non-damaged material.

The intra-yarn orthotropic properties for the current level analysis were based on the refined intra-layer RVE results (Table 5). The calculated effective properties, based on the triply periodic PHFGMC-3D model, exhibit transversely isotropic characteristics consistent with the expected C4 square symmetry, having a principal Young’s modulus of 82.0 GPa and an in-plane shear modulus of 10.8 MPa. These values show good alignment with the linear portion of this material tensile test and Iosipescu shear test results (Figure 12). It also should be noted that the longitudinal Young’s modulus value falls within the upper range of tested tensile modulus values reported for LSI C/C-SiC, spanning from 63 GPa to 83 GPa [6,42,43,44], as expected for the initial undamaged elastic properties.

## 5. Conclusions

This study presents a comprehensive, refined, and adaptive multi-scale framework for modeling the mechanical properties of C/C–SiC composites using the PHFGMC micromechanics formulation. While CT-based microstructural modeling has been reported for C/SiC and SiC/SiC composites, such an approach has not yet been applied to C/C–SiC systems, whose distinctive LSI-induced multi-phase architecture requires a fundamentally different geometric representation. To address this gap, a unique nested PHFGMC strategy was proposed and demonstrated to accurately capture the 8-harness satin weave architecture without introducing additional matrix regions, as these have already been modeled at the micro-level RVE.

- A CT- and SEM-informed hierarchical RVE construction was developed, spanning the fiber/matrix micro-scale, the intra-yarn meso-scale, and the full 8-harness satin ply scale, thereby resolving microstructural features across more than three orders of magnitude in length scale.
- A physically representative geometric description of the woven architecture was achieved using a trapezoidal yarn cross-section approximation, eliminating artificial residual volumes (often interpreted as resin pockets in other composite systems) and enforcing continuous surface contact between interlacing yarns.
- Accurate prediction of effective elastic properties was obtained, with the homogenized stiffness values showing good agreement with the linear regime of tensile and shear experimental data for LSI C/C–SiC composites.
- Refined microstructural RVEs were shown to be essential for local stress-field resolution, as simplified C/C and intra-yarn geometries led to noticeable deviations in transverse stress distributions, highlighting the limitations of idealized representations when local damage-driving fields are of interest.
- A parameter-driven modeling strategy was introduced, providing flexibility to adapt the RVE geometry to different manufacturing conditions, segmentation morphologies, and weave configurations without altering the underlying micromechanical formulation.

Overall, the multi-scale framework developed in this work leverages the computational efficiency of the PHFGMC formulation to deliver valuable insights into the mechanical response of C/C–SiC composites. The refined RVE models derived from advanced material characterization demonstrate clear advantages for accurate stress-field prediction. Consequently, the PHFGMC-CMC framework provides a robust foundation for future extensions that incorporate thermo-mechanical coupling, residual stress analysis, and damage evolution. Finally, the homogenized material properties resulting from the refined CT-informed RVEs can be used to predict the mechanical response of C/C-SiC large-scale structural components.

## Figures and Tables

**Table 1:** In situ mechanical properties of the C/C-RVE constituents [12].

	E_L_, GPa	E_T_, GPa	G_L_, GPa	G_T_, GPa	v_L_	v_T_
Carbon fiber	230.0	13.75	8.96	4.83	0.12	0.25
Porous Carbon matrix	60.68	--	--	--	0.30	--

**Table 2:** PHFGMC predicted mechanical properties for the two C/C-RVEs.

	Refined RVE	Square-Packed Model	
		Value	%Δ (Refined RVE)
E_L_, GPa	185.1	185.4	0%
E_T_, GPa	19.9	21.5	8%
G_L_, GPa	11.7	11.7	0%
G_T_, GPa	7.1	6.5	−8%
v_L_	0.026	0.028	8%
v_T_	0.29	0.25	−16%

**Table 3:** A survey summary of the CT scans listing different characteristics of intra-yarn RVE candidates.

Aspect Ratio	Width/Height	Phase VF	% C/C	% SiC	% Si
Min.	0.63	Min.	68%	9%	1%
Avg.	1.07	Avg.	80%	17%	3%
Max.	1.65	Max.	84%	30%	5%

**Table 4:** In situ mechanical properties of the SiC and free Si complementary phases within the intra-layer RVE [14].

	E_L_, GPa	E_T_, GPa	G_L_, GPa	G_T_, GPa	v_L_	v_T_
SiC	0.001	15.0	5.77	5.77	0.0	0.3
Free Silicon	160	--	--	--	0.22	--

Material properties were adopted from [14], who compiled and adjusted elastic constants originally reported in primary sources (AFML-TR-67-423 and Kelly, 1973).

**Table 5:** Intra-yarn mechanical properties prediction for different geometrical representations.

	E_L_	E_LT_	E_ST_	G_L-ST_	G_L-LT_	G_T_	v_L_^†^	v_T_
	[GPa]	[GPa]	[GPa]	[GPa]	[GPa]	[GPa]	[-]	[-]
Refined RVE	154.1	20.01	20.77	11.23	10.87	7.10	0.03	0.28
Idealized RVE	153.2	20.88	23.09	11.36	10.57	6.51	0.03	0.23
%Δ (Refined RVE)	−1%	4%	11%	1%	−3%	−8%	0%	−17%
W:H = 0.71 model	166.9	20.74	22.42	12.45	11.36	7.31	0.03	0.26
%Δ (Refined RVE)	8%	4%	8%	11%	5%	3%	5%	−4%
W:H = 1.64 model	139.9	19.18	19.36	10.40	10.32	6.97	0.03	0.29
%Δ (Refined RVE)	−9%	−4%	−7%	−7%	−5%	−2%	−2%	5%

^†^—For clarity, Poisson’s modules in the transverse direction were averaged.

## Data Availability

The original contributions presented in this study are included in the article. Further inquiries can be directed to the corresponding author.
